# The active site region plays a critical role in Na^+^ binding to thrombin

**DOI:** 10.1016/j.jbc.2021.101458

**Published:** 2021-11-30

**Authors:** Leslie A. Pelc, Sarah K. Koester, Cassandra R. Kukla, Zhiwei Chen, Enrico Di Cera

**Affiliations:** Edward A. Doisy Department of Biochemistry and Molecular Biology, Saint Louis University School of Medicine, St. Louis, Missouri, USA

**Keywords:** thrombin, protein C, protein conformation, serine protease, enzyme kinetics

## Abstract

The catalytic activity of thrombin and other enzymes of the blood coagulation and complement cascades is enhanced significantly by binding of Na^+^ to a site >15 Å away from the catalytic residue S195, buried within the 180 and 220 loops that also contribute to the primary specificity of the enzyme. Rapid kinetics support a binding mechanism of conformational selection where the Na^+^-binding site is in equilibrium between open (*N*) and closed (*N^∗^*) forms and the cation binds selectively to the *N* form. Allosteric transduction of this binding step produces enhanced catalytic activity. Molecular details on how Na^+^ gains access to this site and communicates allosterically with the active site remain poorly defined. In this study, we show that the rate of the $N^{*}\to N$ transition is strongly correlated with the analogous $E^{*}\to E$ transition that governs the interaction of synthetic and physiologic substrates with the active site. This correlation supports the active site as the likely point of entry for Na^+^ to its binding site. Mutagenesis and structural data rule out an alternative path through the pore defined by the 180 and 220 loops. We suggest that the active site communicates allosterically with the Na^+^ site through a network of H-bonded water molecules that embeds the primary specificity pocket. Perturbation of the mobility of S195 and its H-bonding capabilities alters interaction with this network and influences the kinetics of Na^+^ binding and allosteric transduction. These findings have general mechanistic relevance for Na^+^-activated proteases and allosteric enzymes.

Proteolytic enzymes of the trypsin family utilize the D102/H57/S195 (chymotrypsinogen numbering) triad for activity and participate in physiologically important functions such as digestion, blood coagulation, fibrinolysis, development, fertilization, apoptosis, and immunity (1, 2, 3). These enzymes are synthesized as inactive zymogens to prevent unwanted proteolysis before conversion to the active protease that takes place through a mechanism first proposed by Huber and Bode (4). In this mechanism, the zymogen is cut at the highly conserved residue R15 to generate a new N-terminus that H-bonds to D194 next to the catalytic S195. This prepares the enzyme for efficient substrate binding and catalysis by organizing the primary specificity pocket around D189, structuring the oxyanion hole defined by the backbone N atoms of G193 and S195, and establishing proper H-bonding interactions among residues of the catalytic triad (1, 2, 3, 5).

A few members of the trypsin family of proteases enhance their catalytic activity toward synthetic and physiologic substrates through specific binding of Na^+^ (5, 6). The effect is linked to the evolution of specialized proteases (7, 8), was first observed in enzymes of the blood coagulation cascade (9, 10, 11), and represents one of the most relevant examples of monovalent cation (M^+^) activation in biology (6, 12, 13). In this context, it is important to clarify that the term “activation” refers to the enhancement of catalytic activity induced by M^+^ binding and is distinct from the zymogen to protease conversion of the Huber–Bode mechanism. Some M^+^-activated enzymes utilize the M^+^ as an integral component of the catalytic mechanism and make the M^+^ an absolute requirement for activity. Other enzymes use the M^+^ as an allosteric effector that binds away from the active site and increases activity to the level required by physiologic function. This is the case of the clotting protease thrombin (14) for which Na^+^ is not absolutely required for catalysis, yet it is necessary for efficient procoagulant and signaling activities (15). Binding of Na^+^ to thrombin takes place within a buried site defined by the 180 and 220 loops and located >15 Å away from residues of the catalytic triad (Fig. 1) (16, 17). These loops are organized upon zymogen activation through the Huber–Bode mechanism and not only provide the locale for Na^+^ binding and M^+^ specificity (18, 19), but also define the specificity of the enzyme toward substrate (20, 21). Hence, substrate and Na^+^ binding to thrombin are structurally and functionally linked, but the determinants of such linkage are not fully understood at the molecular level.

**Figure 1:**
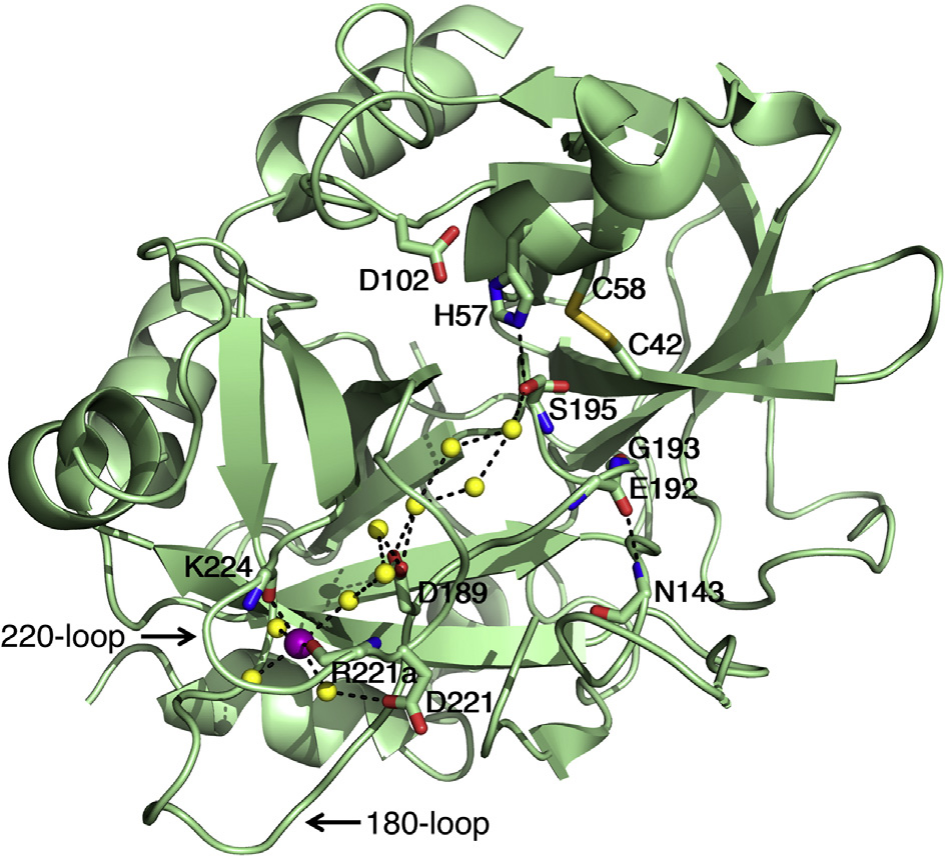
**Ribbon representation of thrombin in the E form bound to Na**^**+**^**(PDB ID:****1SG8****)** (17)**.** The enzyme folds as typically observed for proteases of the trypsin family and is depicted in the Bode orientation, with the active site wide open at the center and the 180 and 220 loops in the south-west corner (60). The residues of the catalytic triad D102/H57/S195 are shown with relevant H-bonds. Na^+^ (*purple*) is bound within these loops and coordinated by four buried water molecules and two backbone O atoms from R221a and K224 (side chains not shown for clarity). D189 at the bottom of the specificity pocket participates indirectly in the coordination shell by supporting one of the waters through H-bonding, a function also played by the side chain of D221. An important backbone H-bond between N143 and E192 (side chains not shown for clarity) stabilizes the orientation of the backbone N atoms of S195 and G193 that define the oxyanion hole responsible for coordination of substrate in the transition state (1, 2, 3). A H-bonded network of water molecules (*yellow*) connects the Na^+^ site to the Oγ atom of S195 across a distance of >15 Å. The position of the C48/C52 disulfide bond next to the catalytic S195 is noted.

The Na-binding affinity is in the mM range, strongly temperature-dependent, and insufficient to saturate the site under physiologic conditions where [Na^+^] = 140 mM (14). Because the [Na^+^] is tightly controlled *in vivo*, the role of Na^+^ is structural rather than regulatory. Residues influencing the energetics of Na^+^ binding at equilibrium have been identified by site-directed mutagenesis and are all located near the Na^+^-binding site, as expected (17). On the other hand, residues responsible for the allosteric transduction of Na^+^ binding into enhanced catalytic activity have been more difficult to identify. Interestingly, the catalytic S195 itself has emerged as a potentially important player in the allosteric transduction (22). The S195T substitution compromises activity because of a potential clash of the bulkier side chain with the adjacent C42/C58 disulfide bond (Fig. 1), as first suggested for trypsin (23), but it also abolishes the Na^+^-induced enhancement of catalytic activity without affecting Na^+^ binding at equilibrium. The same deleterious effect on Na^+^ activation is observed by replacing the active site Ser with Thr in the cognate proteases factor Xa and activated protein C (22). The perturbation also compromises the thrombomodulin-induced enhancement of protein C activation by thrombin and the cofactor Va-induced enhancement of prothrombin activation by factor Xa (22).

In this study, we address the role of S195 and the active site region in Na^+^ activation of thrombin. We identify the likely point of entry of Na^+^ to its site buried within the 180 and 220 loops and show how the properties of residue 195 influence the kinetic mechanism of Na^+^ binding and the resulting allosteric enhancement of catalytic activity through long-range communication with the Na^+^ site.

## Results

### Mechanism of Na^+^ binding

**Figure 2:**
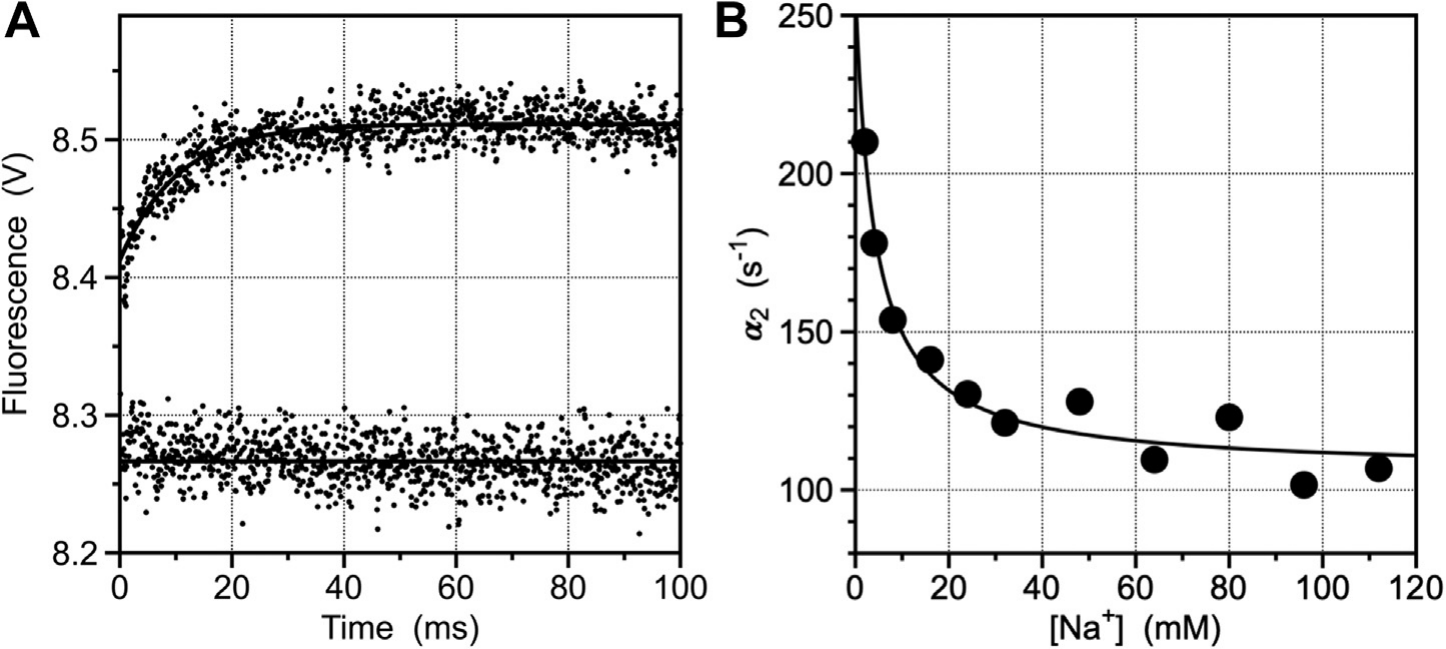
**Rapid kinetics of Na^+^ binding.***A*, kinetic traces of Na^+^ binding to thrombin in the 0 to 100 ms timescale. Shown are the traces obtained at 0 (*bottom trace*) and 50 mM Na^+^ (*top trace*). Binding comprises a fast phase completed within the dead time (0.5 ms) of the stopped flow apparatus, followed by a single-exponential slow phase. Experimental conditions are: 50 nM thrombin, 5 mM Tris, 0.1% PEG, pH 8.0 at 15 °C. The [Na^+^] was changed by keeping the ionic strength constant at 400 mM with ChCl. Continuous lines were drawn using the expression *a* + *be*^*−ct*^, with best-fit parameter values: *a* = 8.5 ± 0.5 V, *b* = 0.099 ± 0.008 V, *c* = 0.096 ± 0.005 ms^−1^. *B*, rate of relaxation for Na^+^ binding to thrombin. The slow relaxation resolved experimentally (*A*) decreases with [Na^+^] and shows that the mechanism of binding obeys conformational selection (Equation 1). Analysis of the slow relaxation according to Equation 2 under the “rapid equilibrium approximation” $k_{off}\gg k_{12}+k_{21}$ gives *α*_2_ (0) = *k*_12_ + *k*_21_ (26). The approximation does not influence resolution of *k*_12_ as the value of the relaxation at large [Na^+^] and provides a lower estimate for the value of *k*_21_. The best-fit parameter values obtained under this approximation are *α*_2_(∞) = *k*_12_ =106 ± 9 s^−1^ and *α*_2_(0) = *k*_12_ = *k*_21_ = 260 ± 20 s^−1^, which gives a lower estimate for *k*_21_ equal to 152 ± 9 s^−1^. In general, the midpoint in the plot is a function of all four independent parameters of Equation 1 (26) and should not be used as a rigorous measure of the *K*_*d*_ for Na^+^ binding. Under the rapid equilibrium approximation, the midpoint does measure *K*_*d*_ as 4.0 ± 0.3 mM. Experimental conditions are: 400 mM ChCl, 50 mM Tris, 0.1% PEG8000, pH 8.0, at 15 °C.

Rapid kinetics of Na^+^ binding to thrombin (24) feature a fast relaxation completed within the dead time (0.5 ms) of the stopped flow apparatus, followed by a slow relaxation consistent with a single exponential phase (Fig. 2*A*). The rate associated with the slow relaxation decreases hyperbolically with [Na^+^] (Fig. 2*B*), and the resulting profile is unequivocal proof of binding according to a mechanism of conformational selection (25, 26), *i.e.*,

(1) $${N^{*}}_{\;}\begin{array}{l} k_{12}\\ ⇄\\ k_{21} \end{array}N\begin{array}{l} k_{on}x\\ ⇄\\ k_{off} \end{array}NX_{\;}$$

Where *x* = [Na^+^]. The mechanism in Equation 1 implies that the Na^+^ site exists in equilibrium between closed, *N^∗^*, and open, *N*, conformations and that Na^+^ (*X*) interacts selectively with *N* through a second-order association rate constant *k*_*on*_ and a first-order dissociation rate constant *k*_*off*_. The equilibrium dissociation constant for Na^+^ binding is defined as the ratio $K_{d}=k_{off}/k_{on}$. Two additional parameters in Equation 1, *k*_12_ and *k*_21_, define the first-order rate constants to open $\left(N^{*}\to N\right)$ and close $\left(N^{*}\leftarrow N\right)$ access to the Na^+^-binding site, respectively. The sum *k*_*eq*_ = *k*_12_ + *k*_21_ measures the rate at which the equilibrium $N^{*}⇌N$ is established (26, 27).

The scheme in Equation 1 contains two independent relaxations, *α*_1_(*x*) and *α*_2_(*x*), reflecting the binding interaction N⇌NX and the conformational transition $N^{*}⇌N$, respectively. Resolution of the four independent rate constants in Equation 1 in the general case demands measurements of both relaxations and analysis through mathematical expressions discussed at length elsewhere (26). In the case of Na^+^ binding to thrombin (Fig. 2*A*), the step N⇌NX reaches equilibrium more rapidly than the $N^{*}⇌N$ step and the relevant expression for *α*_2_(*x*), which is the only relaxation measured experimentally, simplifies as (25, 26)

(2) $$\alpha_{2}\left(x\right)=k_{12}+k_{21}\frac{K_{d}}{K_{d}+x}$$

The expression in Equation 2 contains three independent parameters that can be resolved as the limiting values *α*_2_(0) = *k*_12_ + *k*_21_, *α*_2_(∞) = *k*_12_ and the midpoint of the transition, *K*_*d*_. The value of *k*_12_ =106 ± 9 s^−1^ (Fig. 2*B*) indicates that access to the Na^+^ site opens in <10 ms. The value *k*_21_ =150 ± 20 s^−1^ implies that *k*_*eq*_ = *k*_12_ + *k*_21_ = 260 ± 30 s^−1^ and that the $N^{*}⇌N$ equilibrium is reached in <4 ms. Furthermore, the equilibrium is shifted slightly in favor of the *N∗* conformation (*k*_12_ < *k*_12_), that is populated by 59% of the free molecules in solution.

### Linkage with the $E^{*}⇌E$ equilibrium

**Figure 3:**
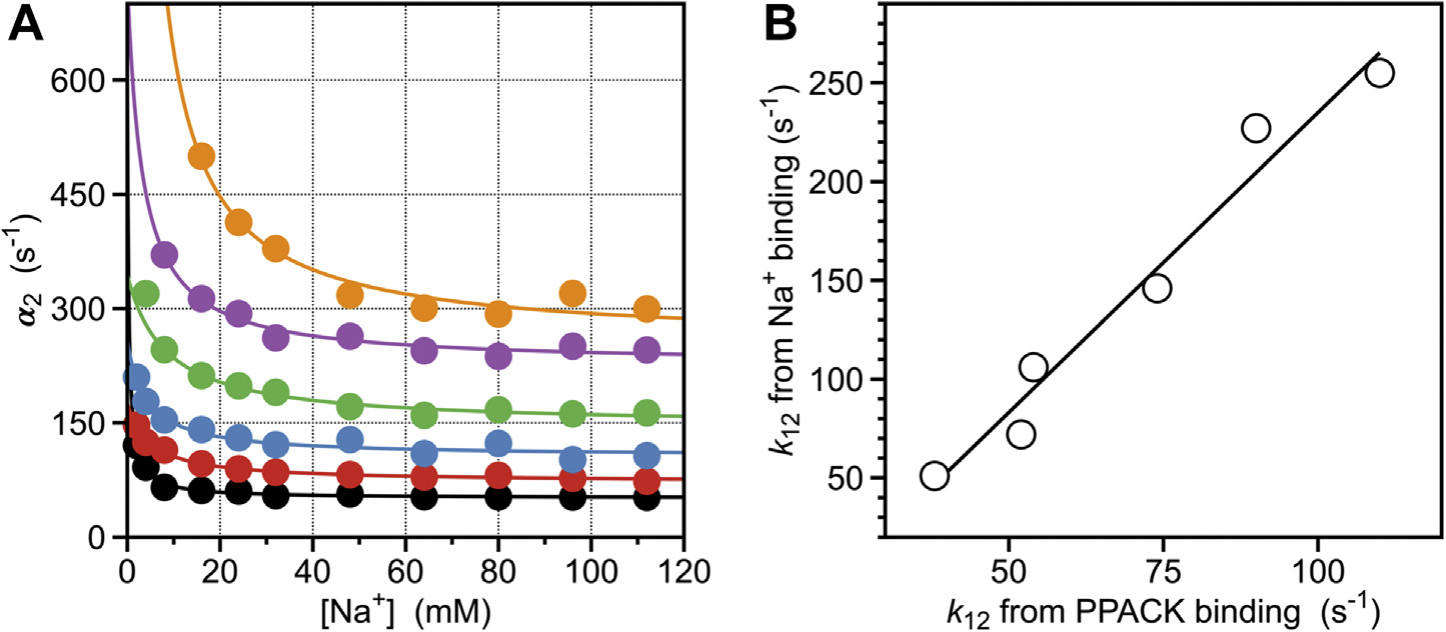
**Temperature dependence of Na^+^ binding.***A*, rate of relaxation for Na^+^ binding to thrombin over the temperature range 5 to 30 °C. *Continuous lines* were drawn under the rapid equilibrium approximation (26), as for the data in Figure 2*B*, with best-fit parameter values listed in Table 1. Experimental conditions are: 400 mM ChCl, 50 mM Tris, 0.1% PEG8000, pH 8.0 at 5 °C (*black*), 10 °C (*red*), 15 °C (*cyan*, see also Fig. 2*A*), 20 °C (*green*), 25 °C (*purple*), 30 °C (*orange*). *B*, values of the rate constant *k*_12_ measuring the $N^{*}\to N$ transition opening access to the Na^+^ site (see panel *A* and Table 1) plotted *versus* the rate constant *k*_12_ measuring the $E^{*}\to E$ transition opening access to the active site, taken from published measurements of PPACK binding under identical solution conditions (44). A strong correlation (*r*^2^ = 0.97) between the two values over the entire temperature range 5 to 30 °C supports a structural linkage between the two binding processes. A van’t Hoff plot of *lnk*_12_*versus*$\frac{1}{T}$ for Na^+^ binding is linear with an activation energy of 11 ± 1 kcal/mol, as found for PPACK binding (44). The continuous line was drawn using the expression *a* + *bx*, with best-fit parameter values: *a* = −68 ± 7 s^−1^, *b* = 3.0 ± 0.2.

The mechanism of Na^+^ binding to thrombin in Equation 1 is reminiscent of the $E^{*}⇌E$ equilibrium between closed (*E^∗^*) and open (*E*) conformations of the active site of trypsin proteases uncovered by structural biology (28, 29) and rapid kinetics (30, 31, 32). The $E^{*}⇌E$ equilibrium refines the Huber–Bode mechanism (33) and explains how activity “spontaneously” emerges in some zymogens (34, 35, 36, 37, 38, 39, 40) or is suppressed in some proteases until interaction with specific cofactors (41, 42). The *E^∗^* form features a small shift of the 215 to 217 segment into the active site that is sufficient to preclude access of substrate to the primary specificity pocket. In general, the *E^∗^* form predominates in the zymogen and is progressively shifted to the *E* form upon transition to the protease, enabling efficient binding and catalysis (32, 33). Importantly, the $E^{*}⇌E$ equilibrium is present in all proteases of the trypsin family, regardless of their ability to bind Na^+^ (28, 29), and indeed Na^+^ binding can be suppressed in Na^+^-activated proteases with little or no effect on the $E^{*}⇌E$ equilibrium (43). Interestingly, the value of *k*_12_ =106 ± 9 s^−1^ measured for Na^+^ binding (Fig. 2*B*) is very similar to the value of *k*_12_ =56 ± 5 s^−1^ measured for binding of the irreversible inhibitor H-D-Phe-Pro-Arg-CH_2_Cl (PPACK) to the active site, under the same experimental conditions (44). The similarity is observed over a broad temperature range (5–30 °C), with a strong correlation (*r*^2^ = 0.97) in the plot where the $N^{*}\to N$ transition opening the Na^+^ binding site (Fig. 3*A* and Table 1) is compared with the $E^{*}\to E$ transition opening the active site (Fig. 3*B*). Nearly identical activation energies (12 kcal/mol) also support a common rate-limiting step and molecular origin for the two processes. We conclude that the $N^{*}⇌N$ equilibrium is a component of the $E^{*}⇌E$ equilibrium and that the active site provides the point of entry for Na^+^ from the bulk solvent to its site buried within the 180 and 220 loops. This conclusion is relevant to all Na^+^-activated proteases in the blood coagulation and complement cascades that share the same locale for Na^+^ binding (3, 5, 6, 45).

**Table 1:** Best-fit parameter values for Na^+^ binding to thrombin (wt) and activated protein C (APC) wild-type and mutants

Protein	T (°C)	*k*_12_ (s^−1^)	*k*_21_ (s^−1^)	*K*_*d*_ (mM)
Wt	5	46 ± 4	94 ± 9	4.3 ± 0.3
Wt	10	72 ± 7	110 ± 10	4.9 ± 0.5
Wt	15	106 ± 9	150 ± 20	4.1 ± 0.4
Wt	20	150 ± 10	260 ± 20	5.0 ± 0.6
Wt	25	230 ± 20	510 ± 40	3.2 ± 0.3
Wt	30	260 ± 20	840 ± 70	6.0 ± 0.5
S195T	15	330 ± 30	550 ± 50	2.8 ± 0.2
C42A/C58A	15	34 ± 3	290 ± 10	3.0 ± 0.3
C42A/C58A/S195T	15	130 ± 10	190 ± 10	3.8 ± 0.3
S195C	15	33 ± 2	270 ± 20	4.0 ± 0.3
APC	15	26 ± 2	74 ± 6	7.0 ± 0.5
APC_180/220_	15	16 ± 2	100 ± 10	7.6 ± 0.5

Analysis based on Equation 2 in the text. Experimental conditions: 20 mM Tris, 200 mM ChCl, 0.1% PEG8000, pH 8.0.

### Role of the 180 and 220 loops

**Figure 4:**
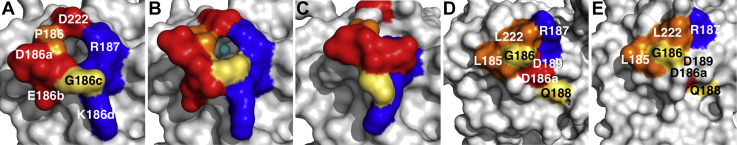
**Region defined by the 180 and 220 loops.***A*–*E*, the crystal structure of thrombin in the *E* form (17), free (*A*) and bound to Na^+^ (*cyan*) (*B*), reveals a pore defined by residues of the 180 and 220 loops that may serve as point of entry to the Na^+^ site. The pore has a pseudoelliptical shape with two axes of 7.3 Å and 5.1 Å that would allow entry of Na^+^ in the fully dehydrated form. Residues are labeled and colored according to their electrostatic properties (*red*, acidic; *blue*, basic; *orange*, hydrophobic; *white*, neutral). The pore closes in the *E^∗^* form (46) (*C*) and is not present in activated protein C (48, 49) (*D*). Swapping the 180 and 220 loops of thrombin with those of activated protein C in Thrombin_180/220_ also occludes the pore (*E*) and does not abolish M^+^ activation (see Fig. 5*B*), proving that the pore is not a point of entry to the Na^+^-binding site.

Support to the foregoing conclusion about the role of the active site as the point of entry for Na^+^ binding comes from mutagenesis of the 180 and 220 loops. The crystal structure of thrombin in the *E* form (17) reveals an intriguing pore defined by the adjacent 180 and 220 loops that could function as a gate to the Na^+^ site (Fig. 4, *A* and *B*). Interestingly, the pore closes in the *E^∗^* form (Fig. 4*C*) when access of substrate to the primary specificity pocket is compromised by a shift of the 215 to 217 segment (46). The linkage between opening and closing of the pore and the $E^{*}⇌E$ equilibrium provides an alternative explanation for the correlation reported in Figure 3*B*. However, this point of entry to the Na^+^ site would make thrombin unique among all Na^+^-activated proteases of the trypsin family because none of them, including factor Xa and activated protein C (45), possess such a pore (47, 48, 49). The role of the 180 and 220 loops in thrombin was studied previously with several replacements, insertions, and deletions and Na^+^ binding was found to be perturbed but not abrogated (18).

**Figure 5:**
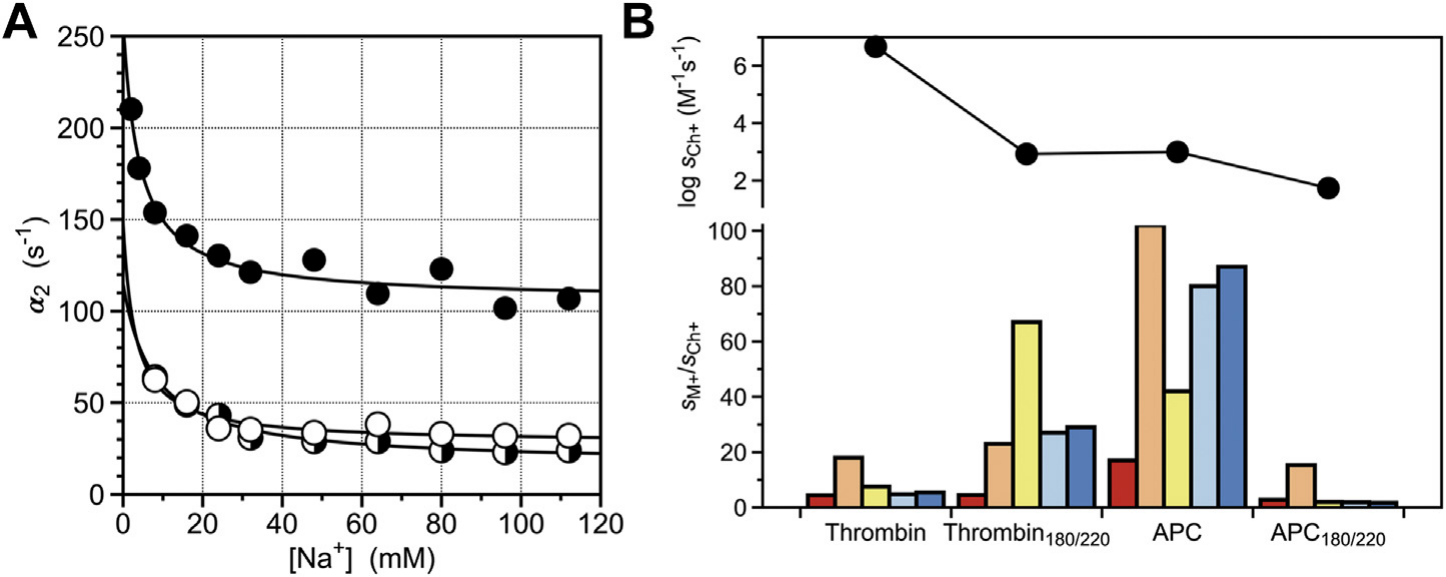
**Loop swap mutants of thrombin and APC.***A*, rate of relaxation for Na^+^ binding to thrombin wild-type (*black*), activated protein C wild-type (*whit*e) and mutant APC_180/220_ (mixed). *Continuous lines* were drawn as for the data in Figure 2*B*, with best-fit parameter values listed in Table 1. Experimental conditions are: 400 mM ChCl, 50 mM Tris, 5 mM EDTA, 0.1% PEG8000, pH 8.0, at 15 °C. *B*, M^+^ activation profile for thrombin wild-type and mutant Thrombin_180/220_, and activated protein C wild-type (APC) and mutant APC_180/220_. Data depict values of the specificity constant $s=k_{\text{cat}}/K_{m}$ (*bottom panel*) for the hydrolysis of chromogenic substrate FPR (thrombin) or S2366 (APC) relative to the value measured in the presence of the inert cation Ch^+^ (*top panel*). M^+^s refer to Li^+^ (*red*), Na^+^ (*orange*), K^+^ (*yellow*), Rb^+^ (*cyan*), Cs^+^ (*blue*). Swapping the 180 and 220 loops between the two enzymes perturbs but does not abolish M^+^ activation. Experimental conditions are: (thrombin) 5 mM Tris, 0.1% PEG8000, pH 8.0 at 25 °C; (APC) 50 mM Tris, 5 mM EDTA, 0.1% PEG8000, pH 8.0 at 25 °C, in the presence of 400 mM Cl^−^ salt as indicated for both thrombin and APC.

Swapping the entire 184a to 188 and 221 to 224 sequences of the 180 and 220 loops between thrombin (^184a^YKPDEGKRG^188^ and ^221^DRDGK^224^) and activated protein C (^184a^ILGDRQ^188^ and ^221^GLLHN^224^) produces a thrombin mutant, Thrombin_180/220_, that is expected to lack the pore (Fig. 4*D*). Indeed, the crystal structure of Thrombin_180/220_ solved at 2.1 Å resolution (Table 2) shows that the pore is occluded (Fig. 4*E*). The mutant features only a rapid jump in fluorescence when Na^+^ binding is studied by stopped flow (data not shown), without the second slow relaxation observed in the wild-type (Fig. 2). The rapid jump is not consistent with loss of Na^+^ binding. In fact, the mutant shows enhanced M^+^ activation and a shift of the M^+^ specificity from Na^+^ to K^+^ (Fig. 5*B*) (19). These structural and functional findings rule out the pore being a gate to the Na^+^ binding site. Further support to this conclusion comes from the reverse swap of the 180 and 220 loops between activated protein C and thrombin, APC_180/220_, that is expected to perturb Na^+^ binding by introducing the pore seen in thrombin (Fig. 4*A*). The APC_180/220_ mutant features a Na^+^-binding profile by rapid kinetics practically identical to that of wild-type activated protein C (Fig. 5*A*), with slightly reduced catalytic activity and M^+^ activation that singles out Na^+^ as an exclusive activator (Fig. 5*B*).

**Table 2:** Crystallographic data for Thrombin_180/220_

PDB entry	7SR9
Buffer/salt	0.1 M Tris, pH 8.5, 0.2 M Li_2_SO_4_
PEG	4000 (30%)
Data collection:	
Wavelength (Å)	1.54
Space group	C2
Unit cell dimensions (Å)	a = 99, b = 78.5, c = 49.6, β = 103.2°
Molecules/asymmetric unit	1
Resolution range (Å)	40–2.1
Observations	63,767
Unique observations	21,066
Completeness (%)	96.9 (86.8)
R_sym_ (%)	6.7 (37.3)
I/σ(I)	14.2 (2.2)
Refinement:	
Resolution (Å)	40–2.1
R_cryst_, R_free_	0.18, 0.22
Reflections (working/test)	19,962/1073
Protein atoms	2336
Solvent molecules	191
Rmsd bond lengths^a^ (Å)	0.010
Rmsd angles^a^ (°)	1.4
Rmsd ΔB (Å^2^) (mm/ms/ss)^b^	1.71/1.61/2.49
<B> protein (Å^2^)	36.1
<B> solvent (Å^2^)	43.0
Ramachandran plot:	
Most favored(%)	100
Generously allowed (%)	0
Disallowed (%)	0

a Root-mean-squared deviation (Rmsd) from ideal bond lengths and angles and Rmsd in B-factors of bonded atoms.

b mm, main chain-main chain; ms, main chain-side chain; ss, side chain-side chain.

### Role of the catalytic S195

**Figure 6:**
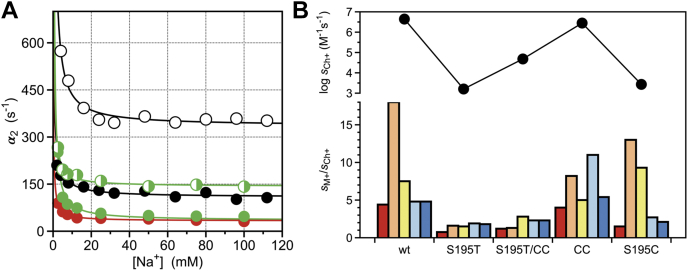
**S195 mutants of thrombin.***A*, rate of relaxation for Na^+^ binding to thrombin wild-type (*black*) and mutants S195T (*white*), C42A/C58A (*green*), C42A/C58A/S195T (*shaded green*), and S195C (*red*). Continuous lines were drawn as for the data in Figure 2*B*, with best-fit parameter values listed in Table 1. Experimental conditions are: 400 mM ChCl, 50 mM Tris, 0.1% PEG8000, pH 8.0, at 15 °C. *B*, M^+^ activation profile for thrombin wild-type and mutants S195T, C42A/C58A (CC), C42A/C58A/S195T (S195T/CC) and S195C. Data depict values of the specificity constant $s=k_{\text{cat}}/K_{m}$ for the hydrolysis of chromogenic substrate FPR (*bottom panel*) relative to the value measured in the presence of the inert cation Ch^+^ (*top panel*). M^+^s refer to Li^+^ (*red*), Na^+^ (*orange*), K^+^ (*yellow*), Rb^+^ (*cyan*), Cs^+^ (*blue*). Experimental conditions are: 5 mM Tris, 0.1% PEG8000, pH 8.0 at 25 °C, in the presence of 400 mM M^+^Cl^−^ salt as indicated.

The foregoing analysis of the potential point of entry of Na^+^ from the bulk solvent to its binding site buried within the 180 and 220 loops draws attention to the long-range communication established by the bound Na^+^ with the active site S195 located >15 Å away (Fig. 1). A recent analysis of the role of S195 as a nucleophile (22) has extended work previously done on the S915T mutant of trypsin (23). Although it is equally represented as Ser in the human genome and is as good a nucleophile, Thr is not documented in the active site of any protease of the trypsin family (22). The recent X-ray structure of the S195T mutant of thrombin shows the methyl group of T195 in direct clash with an incoming substrate and affecting mobility of the reactive Oγ atom, which explains why activity of the S195T mutant is greatly compromised in both thrombin (22) and trypsin (23). The S195T replacement produces additional effects on Na^+^ binding, which is a property not present in trypsin (50). The S195T mutant binds Na^+^ according to Equation 1 (Fig. 6*A*) but is devoid of M^+^ activation (Fig. 6*B*). The value *k*_12_ = 330 ± 30 s^−1^ is significantly faster than that of wild-type, (Table 1) and the value of *k*_21_ = 550 ± 50 s^−1^ implies that the $N^{*}⇌N$ equilibrium is reached within 1.1 ms or four times faster than wild-type. However, these changes do not affect the equilibrium distribution between the two forms because the *N^∗^* conformation is populated by 63% of the free molecules in solution, as seen for wild-type.

Molecular modeling of the S195T mutant of trypsin suggests that the C42/C58 disulfide bond produces steric clash with the bulkier side chain of T195 limiting its mobility within the active site (23). A comparison of the X-ray structures of the S195T mutant of thrombin free and bound to PPACK shows that the Oγ atom of T195 rotates without hindrance within the active site to assume the correct orientation to covalently bind the inhibitor (22). Furthermore, activity of the S195T mutant of trypsin is improved marginally toward amide substrates by removal of the C42/C58 disulfide bond (23). The C42A/C58A replacement in thrombin does not affect activity or M^+^ activation (Fig. 6*B*), but reduces slightly the value of *k*_12_ in the kinetic profile of Na^+^ binding (Fig. 6*A* and Table 1). Interestingly, the C42A/C58A replacement in the S195T background restores the profile of Na^+^ binding and the value of *k*_12_ seen in the wild-type, corrects some of the deficit in catalytic activity but fails to restore M^+^-activation (Fig. 6*B* and Table 1). This shows that the profile of Na^+^ binding resolved by rapid kinetics is influenced by the active site residue and its immediate environment, which is consistent with a remarkable communication taking place across domains separated by >15 Å (Fig. 1). Further support to this conclusion comes from the S195C mutant that replaces the nucleophile with a residue also compatible with catalysis and present in Cys proteases (51). The S195C mutation decreases activity to a level seen for the S195T substitution, but does not abrogate M^+^ activation (Fig. 6*B*). Furthermore, the S195C mutant shows perturbation of the value of *k*_12_ and produces a kinetic profile for Na^+^ binding that is remarkably similar to that of the C42A/C58A mutant (Fig. 6*A* and Table 1).

## Discussion

The results presented in this study address the determinants of the kinetic mechanism of Na^+^ binding to thrombin, a key molecular step for achieving a physiologic level of catalytic activity toward substrates such as fibrinogen and PAR1 (15). The buried nature of the Na^+^ binding site in Na^+^-activated proteases such as thrombin (3, 5, 6, 45, 47, 48, 49) raises the question of how Na^+^ gains access to its site from the bulk solvent. The 180 and 220 loops of thrombin define a pore of pseudoelliptical shape with two relatively short axes of 7.3 Å and 5.1 Å (Fig. 4*A*) (18). Given the ionic radii of Na^+^ (0.9 Å) and water (1.4 Å), the cation must go through the pore in its dehydrated form, as observed in ion transporters (52, 53, 54). The relatively slow rate of association of Na^+^ with thrombin (55) is consistent with a binding step limited by dehydration (56) and makes gating through the pore a potential entry point to the Na^+^ site. However, there is no equivalent of this pore in cognate Na^+^-activated proteases whose Na^+^ binding site is similarly located as in thrombin (3, 5, 47, 48, 49). Mutations, deletions, and insertions of the 180 loop perturb but do not abrogate M^+^ activation (18). Replacing the entire 180 and 220 loops of thrombin with those of activated protein C also produces a mutant that retains M^+^ activation (Fig. 5*B*) even though the pore is no longer present (Fig. 4*D*) (19).

The likely point of entry to the Na^+^ site is provided by the wide aperture of the active site (Fig. 1), which is present in every protease. In this case, the relatively slow rate of association of Na^+^ with thrombin (55) is explained by a dehydration step taking place before entering the active site and/or by displacement of water molecules that embed the primary specificity pocket in a H-bonded network that connects the Na^+^ coordination shell to the reactive Oγ atom of S195 (Fig. 1) (17, 57). The importance of this network became apparent when thrombin was crystallized without ligands in the active site (47, 58) and the Na^+^ site was identified crystallographically (16, 17, 57, 59). Because displacement of waters within the active site is also necessary for substrate and inhibitor binding (60, 61), it is not surprising to observe a significant correlation between the values of *k*_12_ for the $N^{*}\to N$ and $E^{*}\to E$ transitions (Fig. 3*B*). Nearly identical activation energies (12 kcal/mol) for the two processes also vouch for a common rate-limiting step associated with the displacement of waters within the network. Sharing an entry point with substrate would not generate kinetic competition and slow down Na^+^ binding and activation. Ultrarapid kinetics measurements estimate the value of *k*_*on*_ in Equation 1 for Na^+^ binding to be 5.6 × 10^5^ M^−1^ s^−1^ (55). Although this value is more than two orders of magnitude slower than the *k*_*on*_ of substrate binding (22, 30, 62), the physiologic [Na^+^] = 140 mM makes the effective first-order rate *k*_*on*_*x* for the N→NX transition to be several orders of magnitude faster than the value associated with binding of any physiologic or synthetic substrate. Hence, the N⇌NX component of Equation 1 reaches equilibrium within μs, well before any substrate interaction with the active site.

The difference observed in the rates for opening the active site and Na^+^ site (Fig. 3*B*) may not be significant, or it may reflect opening of different sections of the channel defining the primary specificity pocket (17, 57, 60). The wide section at the entrance to the active site enabling access of substrate would open on a slightly slower time scale than the narrow section at the bottom of the specificity pocket giving access to the Na^+^ site (Fig. 1). In either case, the active site would provide the point of entry to the Na^+^ site in thrombin and all Na^+^-activated proteases of the trypsin family. Once bound, Na^+^ communicates long range with the active site S195 through the same network of H-bonded water molecules that need to be displaced transiently during the association step. Subtle changes in the H-bonding connectivity of the waters in the network can be transmitted to the reactive Oγ atom of S195 located >15 Å away and influence the value of *k*_*cat*_ for substrate hydrolysis, as observed experimentally (6, 14, 15, 63). Reciprocally, any perturbation of the nature and mobility of the nucleophile in the active site may affect the kinetic mechanism of Na^+^ binding.

The cross talk between S195 and the Na^+^ site mediated by the water network embedding the primary specificity pocket explains the results reported in this study (Fig. 6). The S195T mutant selectively abrogates M^+^ activation (Fig. 6*B*) without abrogating Na^+^ binding (Fig. 6*A*). The X-ray structure of the S195T mutant in the free form shows a drastically disrupted water network within the active site, even after accounting for the presence of a glycerol molecule from the crystallization buffer (22). The lack of M^+^ activation is not corrected by removal of the adjacent C42/C58 disulfide bond (Fig. 6*B*), unlike the value of *k*_12_ for the $N^{*}\to N$ transition. Repositioning the Oγ atom of T195 and relinquishing its connection with the water network, as documented by the crystal structure (22), would explain the lack of M^+^ activation. Interestingly, the same C42A/C58A replacement in the S195 background also has no effect on M^+^ activation, but reduces the value of *k*_12_ to a level seen in the S195C mutant. C195 is a better nucleophile than S195 and features enough H-bonding strength to ensure proper connectivity with the water network necessary for M^+^ activation. On the other hand, T195 is as good a nucleophile as S195 but lacks the orientation and mobility of its reactive Oγ atom to connect to the water network for M^+^ activation. We conclude that the nature of the nucleophile is directly linked to the value of *k*_12_ for the $N^{*}\to N$ transition and removal of the C42/C58 disulfide bond reduces the value. However, it is the connectivity of the nucleophile with the water network that establishes M^+^ activation.

Consistent with the foregoing conclusion, any perturbation of the water network is expected to interfere with allosteric transduction. Indeed, residue D221 in the 220 loop supports one of the water molecules in the Na^+^ coordination shell (16, 17) and may function as a sensor for Na^+^ binding to initiate allosteric communication (Fig. 1). The D221A mutant abolishes Na^+^ activation by suppressing the transduction step, without affecting Na^+^ binding at equilibrium (17). Half way between the Na^+^ site and the catalytic S195, a critical backbone H-bond between E192 and N143 fixes the architecture of the adjacent oxyanion hole important for substrate binding in the transition state (Fig. 1) (1, 2, 3, 5). As for D221A, the N143P mutant abolishes Na^+^ activation by compromising transduction and without affecting Na^+^ binding at equilibrium (63).

## Experimental procedures

### Protein expression and purification

Thrombin and protein C mutations were generated using the Quik Change Lightning site-directed mutagenesis kit from Agilent Technologies. Appropriate primers synthesized by IDT were used to mutate the residues of the 180 loop in one reaction followed by a second round of mutagenesis to mutate the 220 loop. The thrombin sequences ^184a^YKPDEGKRG^188^ in the 180 loop and ^221^DRDGK^224^ in the 220 loop and the protein C sequences ^184a^ILGDRQ^188^ in the 180 loop and ^221^GLLHN^224^ in the 220 loop were swapped between the two proteases to construct the Thrombin_180/220_ and APC_180/220_ mutants. The primers 5′-AACATGCTGTGTGCGGGCTACAAGCCTGACGAAGGGAAACGAGGGGATGCCTGCGAGGGC-3′ and 5′-AGCTGGGGTGAGGGCTGTGACCGGGATGGGAAATACGGCGTTTACGGCGTTTACACCAAAGTC-3′ were used to replace the 180 and 220 loops of thrombin with those of protein C. The primers 5′-AACATGTTCTGTGCTGGTATCCTCGGGGACCGGCAGGATGCCTGTGAAGGTGAC-3′ and 5′-GGTGAAGGCTGTGGGCTCCTTCACAACTATGGCTTCTAC-3′ were used to replace the 180 and 220 loops of protein C with those of thrombin. Protein constructs were expressed in baby hamster kidney cells using a HPC4-modified pDEST40 (prothrombin) or pRC RSV (protein C) expression vector. Thrombin mutants were expressed and purified as human prothrombin using affinity capture on a HPC4 antibody column followed by ion exchange purification using Q sepharose fast flow and subsequently activated to thrombin using ecarin. Protein C mutants were expressed as previously described (64) and were activated using the thrombin–thrombomodulin complex followed by heparin affinity purification to separate thrombin from activated protein C.

### M^+^ activation

M^+^ activation profiles were calculated from values of the specificity constant $s=k_{\text{cat}}/K_{m}$ for the hydrolysis of the chromogenic substrate H-D-Phe-Pro-Arg-p-nitroanilide (FPR) for thrombin or S2366 (Diapharma) for activated protein C derived from progress curve analysis (65). Experimental conditions were: 5 mM Tris, 0.1% PEG8000, pH 8.0 at 25 °C for thrombin and 50 mM Tris, 5 mM EDTA, 0.1% PEG8000, pH 8.0 at 25 °C for protein C. The salt concentration was 200 mM LiCl, NaCl, KCl, RbCl or CsCl, with choline chloride (ChCl) used as reference, inert M^+^ salt. The M^+^ profile was calculated by expressing the value of $s=k_{\text{cat}}/K_{m}$ for a given M^+^ relative to the value obtained in the presence of Ch^+^.

### Stopped flow

Rapid kinetics experiments were conducted on an Applied Photophysics SX20 stopped-flow spectrometer equipped with an LED light source of the specified wavelength for each experiment, as well as a longpass emission filter. For all experiments, the solution containing the protein diluted to 200 to 500 nM in 50 mM Tris, 400 mM ChCl, 0.1% PEG8000, pH 8.0 at 15 °C was mixed 1:1 with 60 μl of a solution of varying ligand concentration in the same buffer. PPACK, used to probe the active site region, was diluted to varying concentrations in the 2 to 224 μM range in the reaction buffer containing 50 mM Tris, 400 mM ChCl, 0.1% PEG8000, pH 8.0 at 15 °C. Rapid kinetics of PPACK binding to thrombin were studied using an excitation of 295 nm and a cutoff filter at 320 nm. Rapid kinetics of Na^+^ binding to thrombin and activated protein C were studied using an excitation of 280 nm and a cutoff filter at 305 nm. Solutions with varying [Na^+^] in the range 4 to 224 mM were prepared by mixing 400 mM ChCl and 400 mM NaCl in 50 mM Tris, 0.1% PEG8000, pH 8.0 at 15 °C in a ratio to achieve the desired [Na^+^] while keeping the ionic strength of the solution constant. Additionally, 5 mM EDTA was included in experiments with activated protein C to remove the well-established effects of Ca^2+^ in promoting Na^+^ binding (49, 66, 67). Baselines were measured by mixing thrombin or activated protein C into buffer in the absence of ligand. Each kinetic trace for a given ligand concentration was taken as the average of a minimum of six determinations. The traces were fit to a single exponential using software supplied by Applied Photophysics. For all experiments, the final protein concentration in the reaction cell varied from 100 to 250 nM. Data for Na^+^ binding to thrombin in the temperature range from 5 to 30 °C were obtained by titrating the buffer to pH 8.0 using the appropriate temperature coefficient for Tris (68).

### X-ray studies

Crystallization of Thrombin_180/220_ was achieved at 22 °C by the vapor diffusion technique, with each crystallization reservoir containing 500 μl of solution. Equal volumes of the protein sample with 7.4 mg/ml and reservoir solution (2 μl each) were mixed to prepare the hanging drops. Crystals were grown in the presence of 0.1 M Tris, pH 8.5, 0.2 M Li_2_SO_4_, 30% PEG4000 in 2 weeks. Diffraction quality crystals were frozen using 15% glycerol as cryoprotectant at 100° K with a home source (Rigaku 1.2 kw MMX007 generator with VHF optics) Rigaku Raxis IV++ detector and were indexed, integrated and scaled with the HKL2000 software package (69). The structure was solved by molecular replacement using MOLREP from the CCP4 suite (70) and Protein Data Bank accession code 1SHH as a search model. Refinement and electron density generation were done using REFMAC5 from the CCP4 package. Five percent of the reflections were randomly selected as a test set for cross-validation. Model building and analysis were carried out using COOT (71). Ramachandran plots were calculated using PROCHECK (72). Statistics for data collection and refinement are summarized in Table 2. Atomic coordinates and structure factors have been deposited in Protein Data Bank (PDB ID: 7SR9).

## Data availability

All data described in the manuscript are contained within the manuscript.

## Conflict of interest

The authors declare no conflict of interest with the content of this article.

## References

[1] Perona J.J., Craik C.S. (1995). Structural basis of substrate specificity in the serine proteases. Protein Sci..

[2] Hedstrom L. (2002). Serine protease mechanism and specificity. Chem. Rev..

[3] Page M.J., Di Cera E. (2008). Serine peptidases: Classification, structure and function. Cell. Mol. Life Sci..

[4] Huber R., Bode W. (1978). Structural basis of the activation and action of trypsin. Acc. Chem. Res..

[5] Di Cera E. (2009). Serine proteases. IUBMB Life.

[6] Page M.J., Di Cera E. (2006). Role of Na+ and K+ in enzyme function. Physiol. Rev..

[7] Krem M.M., Di Cera E. (2001). Molecular markers of serine protease evolution. EMBO J..

[8] Krem M.M., Di Cera E. (2002). Evolution of enzyme cascades from embryonic development to blood coagulation. Trends Biochem. Sci..

[9] Orthner C.L., Kosow D.P. (1980). Evidence that human alpha-thrombin is a monovalent cation-activated enzyme. Arch. Biochem. Biophys..

[10] Steiner S.A., Amphlett G.W., Castellino F.J. (1980). Stimulation of the amidase and esterase activity of activated bovine plasma protein C by monovalent cations. Biochem. Biophys. Res. Commun..

[11] Orthner C.L., Kosow D.P. (1978). The effect of metal ions on the amidolytic acitivity of human factor Xa (activated Stuart-Prower factor). Arch. Biochem. Biophys..

[12] Di Cera E. (2006). A structural perspective on enzymes activated by monovalent cations. J. Biol. Chem..

[13] Gohara D.W., Di Cera E. (2016). Molecular mechanisms of enzyme activation by monovalent cations. J. Biol. Chem..

[14] Wells C.M., Di Cera E. (1992). Thrombin is a Na(+)-activated enzyme. Biochemistry.

[15] Di Cera E. (2008). Thrombin. Mol. Aspects Med..

[16] Di Cera E., Guinto E.R., Vindigni A., Dang Q.D., Ayala Y.M., Wuyi M., Tulinsky A. (1995). The Na+ binding site of thrombin. J. Biol. Chem..

[17] Pineda A.O., Carrell C.J., Bush L.A., Prasad S., Caccia S., Chen Z.W., Mathews F.S., Di Cera E. (2004). Molecular dissection of Na+ binding to thrombin. J. Biol. Chem..

[18] Prasad S., Wright K.J., Roy D.B., Bush L.A., Cantwell A.M., Di Cera E. (2003). Redesigning the monovalent cation specificity of an enzyme. Proc. Natl. Acad. Sci. U. S. A..

[19] Rana S., Pozzi N., Pelc L.A., Di Cera E. (2011). Redesigning allosteric activation in an enzyme. Proc. Natl. Acad. Sci. U. S. A..

[20] Hedstrom L., Farr-Jones S., Kettner C.A., Rutter W.J. (1994). Converting trypsin to chymotrypsin: Ground-state binding does not determine substrate specificity. Biochemistry.

[21] Hedstrom L., Szilagyi L., Rutter W.J. (1992). Converting trypsin to chymotrypsin: The role of surface loops. Science.

[22] Pelc L.A., Chen Z., Gohara D.W., Vogt A.D., Pozzi N., Di Cera E. (2015). Why Ser and not Thr brokers catalysis in the trypsin fold. Biochemistry.

[23] Baird T.T., Wright W.D., Craik C.S. (2006). Conversion of trypsin to a functional threonine protease. Protein Sci..

[24] Bah A., Garvey L.C., Ge J., Di Cera E. (2006). Rapid kinetics of Na+ binding to thrombin. J. Biol. Chem..

[25] Vogt A.D., Di Cera E. (2012). Conformational selection or induced fit? A critical appraisal of the kinetic mechanism. Biochemistry.

[26] Di Cera E. (2020). Mechanisms of ligand binding. Biophys. Rev..

[27] Fersht A.R. (1999).

[28] Gohara D.W., Di Cera E. (2011). Allostery in trypsin-like proteases suggests new therapeutic strategies. Trends Biotechnol..

[29] Pozzi N., Vogt A.D., Gohara D.W., Di Cera E. (2012). Conformational selection in trypsin-like proteases. Curr. Opin. Struct. Biol..

[30] Vogt A.D., Chakraborty P., Di Cera E. (2015). Kinetic dissection of the pre-existing conformational equilibrium in the trypsin fold. J. Biol. Chem..

[31] Vogt A.D., Pozzi N., Chen Z., Di Cera E. (2014). Essential role of conformational selection in ligand binding. Biophys. Chem..

[32] Chakraborty P., Acquasaliente L., Pelc L.A., Di Cera E. (2018). Interplay between conformational selection and zymogen activation. Sci. Rep..

[33] Stojanovski B.M., Chen Z., Koester S.K., Pelc L.A., Di Cera E. (2019). Role of the I16-D194 ionic interaction in the trypsin fold. Sci. Rep..

[34] Yamamoto E., Kitano Y., Hasumi K. (2011). Elucidation of crucial structures for a catechol-based inhibitor of plasma hyaluronan-binding protein (factor VII activating protease) autoactivation. Biosci. Biotechnol. Biochem..

[35] Sichler K., Banner D.W., D'Arcy A., Hopfner K.P., Huber R., Bode W., Kresse G.B., Kopetzki E., Brandstetter H. (2002). Crystal structures of uninhibited factor VIIa link its cofactor and substrate-assisted activation to specific interactions. J. Mol. Biol..

[36] Whitcomb D.C., Gorry M.C., Preston R.A., Furey W., Sossenheimer M.J., Ulrich C.D., Martin S.P., Gates L.K., Amann S.T., Toskes P.P., Liddle R., McGrath K., Uomo G., Post J.C., Ehrlich G.D. (1996). Hereditary pancreatitis is caused by a mutation in the cationic trypsinogen gene. Nat. Genet..

[37] Stirnberg M., Maurer E., Horstmeyer A., Kolp S., Frank S., Bald T., Arenz K., Janzer A., Prager K., Wunderlich P., Walter J., Gutschow M. (2010). Proteolytic processing of the serine protease matriptase-2: Identification of the cleavage sites required for its autocatalytic release from the cell surface. Biochem. J..

[38] Shamanaev A., Emsley J., Gailani D. (2021). Proteolytic activity of contact factor zymogens. J. Thromb. Haemost..

[39] Pozzi N., Chen Z., Zapata F., Niu W., Barranco-Medina S., Pelc L.A., Di Cera E. (2013). Autoactivation of thrombin precursors. J. Biol. Chem..

[40] Pozzi N., Barranco-Medina S., Chen Z., Di Cera E. (2012). Exposure of R169 controls protein C activation and autoactivation. Blood.

[41] Edgington T.S., Mackman N., Brand K., Ruf W. (1991). The structural biology of expression and function of tissue factor. Thromb. Haemost..

[42] Forneris F., Ricklin D., Wu J., Tzekou A., Wallace R.S., Lambris J.D., Gros P. (2010). Structures of C3b in complex with factors B and D give insight into complement convertase formation. Science.

[43] Niu W., Chen Z., Gandhi P.S., Vogt A.D., Pozzi N., Pelc L.A., Zapata F.J., Di Cera E. (2011). Crystallographic and kinetic evidence of allostery in a trypsin-like protease. Biochemistry.

[44] Pelc L.A., Koester S.K., Chen Z., Gistover N.E., Di Cera E. (2019). Residues W215, E217 and E192 control the allosteric E∗-E equilibrium of thrombin. Sci. Rep..

[45] Vogt A.D., Bah A., Di Cera E. (2010). Evidence of the E∗-E equilibrium from rapid kinetics of Na(+) binding to activated protein C and factor Xa. J. Phys. Chem. B.

[46] Pineda A.O., Chen Z.W., Bah A., Garvey L.C., Mathews F.S., Di Cera E. (2006). Crystal structure of thrombin in a self-inhibited conformation. J. Biol. Chem..

[47] Zhang E., Tulinsky A. (1997). The molecular environment of the Na+ binding site of thrombin. Biophys. Chem..

[48] Mather T., Oganessyan V., Hof P., Huber R., Foundling S., Esmon C., Bode W. (1996). The 2.8 A crystal structure of Gla-domainless activated protein C. Embo J..

[49] Schmidt A.E., Padmanabhan K., Underwood M.C., Bode W., Mather T., Bajaj S.P. (2002). Thermodynamic linkage between the S1 site, the Na+ site, and the Ca2+ site in the protease domain of human activated protein C (APC). Sodium ion in the APC crystal structure is coordinated to four carbonyl groups from two separate loops. J. Biol. Chem..

[50] Dang Q.D., Di Cera E. (1996). Residue 225 determines the Na(+)-induced allosteric regulation of catalytic activity in serine proteases. Proc. Natl. Acad. Sci. U. S. A..

[51] Van Opdenbosch N., Lamkanfi M. (2019). Caspases in cell death, inflammation, and disease. Immunity.

[52] Doyle D.A., Morais Cabral J., Pfuetzner R.A., Kuo A., Gulbis J.M., Cohen S.L., Chait B.T., MacKinnon R. (1998). The structure of the potassium channel: Molecular basis of K+ conduction and selectivity. Science.

[53] Roux B., MacKinnon R. (1999). The cavity and pore helices in the KcsA K+ channel: Electrostatic stabilization of monovalent cations. Science.

[54] Zhou Y., MacKinnon R. (2004). Ion binding affinity in the cavity of the KcsA potassium channel. Biochemistry.

[55] Gianni S., Ivarsson Y., Bah A., Bush-Pelc L.A., Di Cera E. (2007). Mechanism of Na+ binding to thrombin resolved by ultra-rapid kinetics. Biophys. Chem..

[56] Eigen M. (1957). Determination of general and specific ionic interactions in solution. Discuss. Faraday Soc..

[57] Krem M.M., Di Cera E. (1998). Conserved water molecules in the specificity pocket of serine proteases and the molecular mechanism of Na+ binding. Proteins.

[58] Rydel T.J., Tulinsky A., Bode W., Huber R. (1991). Refined structure of the hirudin-thrombin complex. J. Mol. Biol..

[59] Guinto E.R., Caccia S., Rose T., Futterer K., Waksman G., Di Cera E. (1999). Unexpected crucial role of residue 225 in serine proteases. Proc. Natl. Acad. Sci. U. S. A..

[60] Bode W., Turk D., Karshikov A. (1992). The refined 1.9-A X-ray crystal structure of D-Phe-Pro-Arg chloromethylketone-inhibited human alpha-thrombin: Structure analysis, overall structure, electrostatic properties, detailed active-site geometry, and structure-function relationships. Protein Sci..

[61] Malikayil J.A., Burkhart J.P., Schreuder H.A., Broersma R.J., Tardif C., Kutcher L.W., Mehdi S., Schatzman G.L., Neises B., Peet N.P. (1997). Molecular design and characterization of an alpha-thrombin inhibitor containing a novel P1 moiety. Biochemistry.

[62] Chakraborty P., Di Cera E. (2017). Induced fit is a special case of conformational selection. Biochemistry.

[63] Niu W., Chen Z., Bush-Pelc L.A., Bah A., Gandhi P.S., Di Cera E. (2009). Mutant N143P reveals how Na+ activates thrombin. J. Biol. Chem..

[64] Stojanovski B.M., Pelc L.A., Di Cera E. (2020). Role of the activation peptide in the mechanism of protein C activation. Sci. Rep..

[65] Krem M.M., Di Cera E. (2003). Dissecting substrate recognition by thrombin using the inactive mutant S195A. Biophys. Chem..

[66] Schmidt A.E., Stewart J.E., Mathur A., Krishnaswamy S., Bajaj S.P. (2005). Na+ site in blood coagulation factor IXa: Effect on catalysis and factor VIIIa binding. J. Mol. Biol..

[67] Underwood M.C., Zhong D., Mathur A., Heyduk T., Bajaj S.P. (2000). Thermodynamic linkage between the S1 site, the Na+ site, and the Ca2+ site in the protease domain of human coagulation factor xa. Studies on catalytic efficiency and inhibitor binding. J. Biol. Chem..

[68] Ayala Y.M., Di Cera E. (2000). A simple method for the determination of individual rate constants for substrate hydrolysis by serine proteases. Protein Sci..

[69] Otwinowski Z., Minor W. (1997). Processing of x-ray diffraction data collected by oscillation methods. Methods Enzymol..

[70] Bailey S. (1994). The CCP4 suite. Programs for protein crystallography. Acta Crystallogr. D Biol. Crystallogr..

[71] Emsley P., Cowtan K. (2004). Coot: Model-building tools for molecular graphics. Acta Crystallogr. D Biol. Crystallogr..

[72] Morris A.L., MacArthur M.W., Hutchinson E.G., Thornton J.M. (1992). Stereochemical quality of protein structure coordinates. Proteins.

